# Adaptive Prediction As a Strategy in Microbial Infections

**DOI:** 10.1371/journal.ppat.1004356

**Published:** 2014-10-02

**Authors:** Sascha Brunke, Bernhard Hube

**Affiliations:** 1 Department of Microbial Pathogenicity Mechanisms, Leibniz Institute for Natural Product Research and Infection Biology, Hans Knöll Institute (HKI), Jena, Germany; 2 Center for Sepsis Control and Care, Jena University Hospital, Jena, Germany; 3 Friedrich Schiller University, Jena, Germany; Duke University Medical Center, United States of America

Microorganisms need to sense and respond to constantly changing microenvironments, and adapt their transcriptome, proteome, and metabolism accordingly to survive [1]. However, microbes sometimes react in a way which does not make immediate biological sense in light of the current environment—for example, by up-regulating an iron acquisition system in times of metal abundance. The reason for this seemingly nonsensical behavior can lie in the microbe's ability to predict a coming change in conditions by cues from the current environment. If the microbe (pre-)adapts accordingly, it will increase its fitness and chances of survival under subsequent selection pressures—a concept known as adaptive prediction (Figure 1) [2].

**Figure 1:**
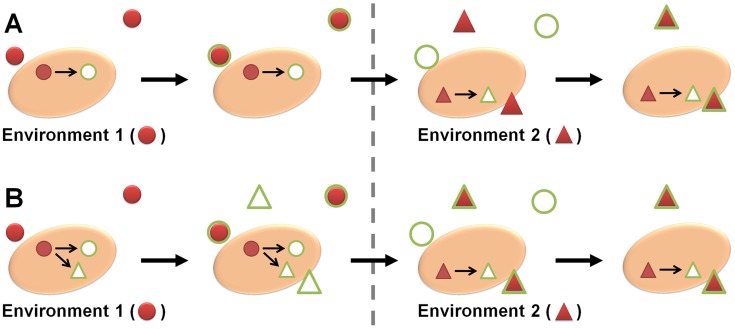
The basis of adaptive prediction. (A) The conditions in environment 1 (red circles) activate a distinct response (green circles) in a microbe. After changing to environment 2, altering, e.g., the expression pattern to respond to the new conditions (triangles) requires time, during which the microbe is not well adapted. (B) If the sequential temporal order of the two environments from (A) is kept over many generations, a new signal pathway can form. Now, the conditions in environment 1 induce responses to both the first and the second environment. When changing to environment 2, the microbe is hence already pre-adapted.

In metazoans with complex neural network architecture, the capacity to anticipate changes in the environment is understandable. It can be achieved in a single multicellular organism, e.g., by classical conditioning. In unicellular organisms, however, this type of learning normally requires generations of selection pressure to connect one predictor to a coming condition.

## Why Is Adaptive Prediction Relevant for Human Pathogens?

The human host is, to a certain extent, a highly predictable environment. In its different niches, pH values, ion concentrations, temperature, and many other factors are normally kept within small ranges. Transiting from one niche to another usually follows a predetermined pattern—entering the host from the environment is associated with an increase in temperature; in the gastrointestinal (GI) tract, the neutral gut will follow the strongly acidic stomach; invasion into tissue and entering the bloodstream will likely lead to engulfment by immune cells, followed by oxidative stress and starvation for micronutrients such as iron or zinc; and passaging through the gut means decreasing oxygen and glucose levels. These cues can be used by commensals and potential pathogens to optimize their fitness by predicting the next stage in host–microbe interaction.

## Sensing and Making Sense—The Example of *Escherichia coli* and Other Enteric Bacteria

A good example for adaptive prediction comes from the gut bacterium *Escherichia coli*. In this microbe, an increase in temperature elicits a transcriptional response typical for low oxygen levels [3]. This makes biological sense, as the increase in temperature can indicate the bacterium's arrival in the gut, where oxygen will soon become limiting. Interestingly, this predictive function can be disrupted if temperature and oxygen levels are dissociated over evolutionary timescales. In a laboratory microevolution experiment with a reversed temperature–oxygen relationship (i.e., high temperature is followed by high oxygen), Tagkopoulos et al. obtained *E. coli* strains where the predictive quality of temperature for oxygen was largely lost [3]. Similarly, maltose utilization genes are activated in *E. coli* upon exposure to lactose, reflecting the sequential abundance of these sugars in the gut [2]. Again, disruption of this sequence over hundreds of generations was able to abolish this adaptive prediction in vitro [2]. These two examples show how strongly an evolved adaptive prediction response can impact microbial fitness.

As many pathogens are gut-associated, similar patterns can be found in pathogenic enteric bacteria. The enterohemorrhagic *E. coli* (EHEC) serotype O157:H7, for example, can use the presence of bile as a signal to induce transcription of iron acquisition genes, independent of actual iron levels [4]. This can be useful in the iron-sequestering environment of the small intestine, where bile abounds. On the other hand, pathogenicity-island encoded genes that are specifically expressed at later stages of the intestinal passage by EHECs were found to be repressed by bile in the upper part of the small intestine [4]. Many other enteric bacteria, like *Salmonella*, *Shigella*, and *Vibrio* spp. also use bile as a signal to regulate virulence programs, which are biologically unlinked to bile salts but are advantageous at later stages in their mammalian hosts (reviewed in [5]). *Vibrio cholerae* is also known to induce genes late in its infection cycle that are of no immediate use in the host. These genes, for example those involved in chitin binding and degradation, should benefit the bacteria only after they are released into the aquatic environment where crustaceans provide ample chitin [6]—although it is tempting to speculate that chitin degradation may play an additional role in competition with resident fungi in the gut. In summary, sensing certain host-specific factors can herald changing conditions, and pathogens can use these signals in their (pre-)adaptation to the host or for transition from the host.

## (Re-)interpreting Old Cues—The *Candida albicans* Example

**Figure 2:**
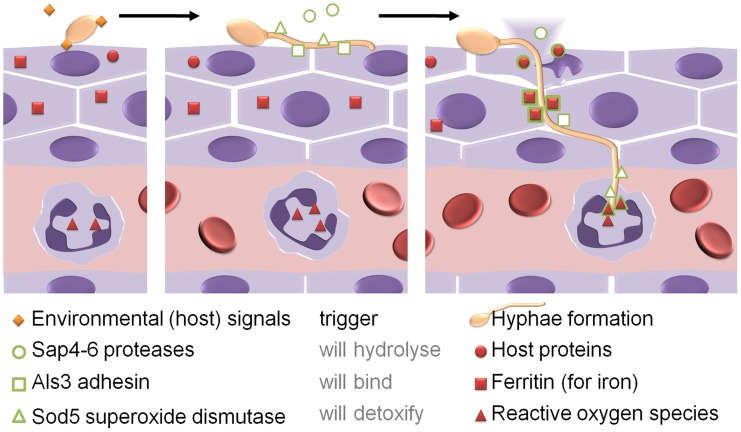
*Candida albicans* as an example for adaptive prediction of pathogens in the host. When attaching to epithelial cells, environmental signals trigger hyphae formation. The hyphae start to express a set of proteins which are not apparently beneficial for the fungus in its current situation (green symbols). Only when penetrating into the host tissue and during encounters with host immune cells like neutrophils, the stresses (red symbols) occur under which these proteins give *C. albicans* an advantage in survival and growth (see text for more details).

*Candida albicans* is a fungal pathogen that can transit from a commensal state in the gut to an aggressive pathogen that invades tissue and disseminates via the bloodstream. Tissue invasion is linked to a specific morphology change, the yeast-to-hypha transition (Figure 2). The hyphal program is triggered by multiple stimuli, including contact with epithelial cells and body temperature [7]. Part of this program is the expression of the multipurpose, hypha-associated cell wall protein, Als3, which enables the fungus to attach to and invade host cells and use the intracellular host iron storage protein ferritin as an iron source after invasion [8]. Other hypha-associated factors are the Sap4-6 proteases, which can degrade host proteins during invasion, and the cell surface localized superoxide dismutase Sod5, which can be used to detoxify reactive oxygen species likely to be produced by attracted immune effector cells when tissue is damaged. Therefore, by triggering hyphal morphogenesis, *C. albicans* produces factors that are required during or after tissue invasion even before the actual invasion process is initiated [7].

In the blood, *C. albicans* seems to interpret the presence of (even low) amounts of glucose as a cue for potential encounters with immune cells. While the related baker's yeast, *Saccharomyces cerevisiae*, down-regulates most stress-response genes in the presence of glucose, *C. albicans* up-regulates oxidative and osmotic stress responses when encountering glucose levels similar to the bloodstream [9]. While not necessary for growth on glucose, these adaptations would allow better survival of attacks by blood-borne phagocytic cells after leaving the glucose-poor gut. Accordingly, the signaling networks leading from glucose to stress response differ significantly between the two species, allowing *C. albicans* to reinterpret glucose as a pre-indicator of possible future dangers [9].

In a similar vein, *C. albicans* responds to neutral or alkaline pH by expressing genes involved in iron and zinc uptake via an alkaline-induced transcription factor, Rim101 [10], [11]. As these metals are generally less soluble at high pH, this connection makes biological sense and may help in a timely response, even before the full effect of metal limitation is felt by the cell. Thus, common environmental cues like presence of carbon sources or pH changes can obtain a new, additional meaning and allow the pathogen to predict conditions in different host niches. To this end, established signaling pathways for these conditions can be rewired to novel outputs and thus allow an adaptive prediction response.

## Weighing Costs and Risks—The *Plasmodium* Example

Predicting the future environment comes with a risk. A “false positive” prediction—in which the pathogen falsely predicts a future environment that it will not encounter in reality—will leave the pathogen in a state less adapted to the current environment, with all the associated fitness costs. A “false negative” prediction (in which the pathogen does not interpret the signal correctly to prepare for a future change) will lead to a severe loss in fitness in the coming environment [12]. Because of this trade-off, any (costly) adaptation must rely on robust and reliable signals before a population of cells commits to a new phenotype. Alternatives exist in the form of stochastic switching and phenotypic heterogeneity, in which only a random subpopulation expresses a certain trait [13]. This strategy is more common in unpredictable and fluctuating environments [14].

The causative agents of malaria, *Plasmodium* spp., normally replicate asexually inside the bloodstream of their host. However, at every replication cycle, a portion of the parasites develops into gametocytes instead. For these sexual stages, the mammalian host is a dead end, as the gametocytes cannot replicate asexually anymore. However, after a mosquito bite, only gametocytes can enter this new, suitable host to differentiate and mate [15]. Thus, in every replication cycle, there is a trade-off between investing resources into forming the sexual stage for propagation between hosts and asexual reproduction within a host. Interestingly, the rate of conversion to the sexual stage varies between *Plasmodium* species, and antimalarial treatment, as well as an increase in young reticulocytes, increases the number of sexual gametocytes [16]. The malaria parasites use these indicators as signs of imminent host death or clearance of infection. In a “terminal investment,” the sexual between-host transmission strategy is then followed. Similarly, in a freshly infected naive host, investment in sexual forms is possible since the associated fitness costs are low. In contrast, in the presence of low levels of stress, for example caused by parasites of different genotypes competing for the same host resources, fitness costs for not replicating asexually are high, and asexual reproduction, hence, dominates (discussed in [17]). Overall, environmental cues allow the pathogen to weigh the risks for committing to a pre-adapted phenotype.

## Adaptive Prediction and Coordinated Regulation

Adaptive prediction seems, in many aspects, similar to the concept of coordinate regulation, in which several genes, often including virulence factors, are controlled by a common regulatory system in response to an environmental trigger [18]. Conceptually, however, coordinate regulation responds to environmental factors that are linked by their simultaneous occurrence rather than their temporal succession. A good example is the iron-starvation–induced expression of the siderophore synthesis machinery, siderophore binding proteins, and cytolytic toxins in many bacteria. In that process, iron starvation indicates a host environment or activities by the host, and a coordinated transcriptional regulation allows immediate destruction of host cells, binding, and finally, uptake of iron in response. In a sense, signal and bacterial adaptation responses are spatially linked, as they occur in the immediate environment of the microbe. In contrast, in adaptive prediction, signal and responses are temporally linked.

It may prove difficult, however, to draw a precise dividing line between the two concepts, as many intermediate forms likely exist. Furthermore, a coordinated regulation could feasibly evolve into an adaptive prediction system. Coordinately regulated genes come under control of one or a few transcription factors or regulatory pathways. If an independent signal (nearly) always predictively precedes the coordinated expression, these few signal pathways (or the single pathway) can easily evolve to accept this signal for a “pre-emptive” response [3]. Adding a predictive to the existing immediate trigger, hence, allows a complex and fully coordinated response to take place in anticipation of a new environment. This way, coordinated regulation could make the appearance of adaptive prediction evolutionary more likely.

On the other hand, the expression of many genes can come with a higher fitness cost. Mathematical models show that this kind of adaptive prediction is more likely to occur in environments where stresses (rather than future improvement in growth conditions) are able to be predicted well and may be even modified to include a partial response (for details, see [12]).

## Prevalence and Possible Medical Applications of Adaptive Prediction

How prevalent is this phenomenon in pathogens? It seems likely that adaptive prediction processes are more common than is currently appreciated. In the laboratory, microbes are rarely exposed to two or more consecutive environments that reflect the natural progression through habitats. Unusual (i.e., predictive) transcriptional responses occur, but without a biological explanation these may not be followed up when investigating the microbe's response to a specific environment. Especially in environmental microbes, which are not known to be generally associated with animal hosts, a host-adaptive response to certain environmental stresses may indicate potential for pathogenicity. Such adaptations would likely be different to commensal organisms, and may result from transient but repeated exposure to animal hosts.

In simulations, predictive behavior of genetic networks appears fast and frequently [3]. In directed evolution experiments, yeast can acquire the ability to predict one stress from the presence of another remarkably quickly [19]. Finally, without the need for evolutionary processes spanning generations, associative learning is considered feasible in individual single cell organisms [20] and even in simple chemical networks [21]. While still mostly hypothetical, this would allow microbes to expand beyond evolutionarily acquired adaptive prediction into responses shaped by individual cell life histories.

It therefore seems highly likely that many pathogens can switch to a (currently) non-adaptive phenotype when external cues indicate a coming change in environment. Using these signals to “trick” a pathogen into a phenotypic conversion may be exploited to render microbes maladapted to their current surroundings. As an avenue for future treatment options, adaptive prediction responses may therefore deserve deeper consideration.
